# Mitogenomic Evidence for an Indo-West Pacific Origin of the Clupeoidei (Teleostei: Clupeiformes)

**DOI:** 10.1371/journal.pone.0056485

**Published:** 2013-02-19

**Authors:** Sébastien Lavoué, Masaki Miya, Prachya Musikasinthorn, Wei-Jen Chen, Mutsumi Nishida

**Affiliations:** 1 Institute of Oceanography, National Taiwan University, Taipei, Taiwan; 2 Atmosphere and Ocean Research Institute, The University of Tokyo, Chiba, Japan; 3 Natural History Museum and Institute, Chiba, Japan; 4 Faculty of Fisheries, Kasetsart University, Bangkok, Thailand; University of Bologna, Italy

## Abstract

The clupeoid fishes are distributed worldwide, with marine, freshwater and euryhaline species living in either tropical or temperate environments. Regional endemism is important at the species and genus levels, and the highest species diversity is found in the tropical marine Indo-West Pacific region. The clupeoid distribution follows two general pattern of species richness, the longitudinal and latitudinal gradients. To test historical hypotheses explaining the formation of these two gradients, we have examined the early biogeography of the Clupeoidei in reconstructing the evolution of their habitat preferences along with their ancestral range distributions on a time-calibrated mitogenomic phylogeny. The phylogenetic results support the distinction of nine main lineages within the Clupeoidei, five of them new. We infer several independent transitions from a marine to freshwater environment and from a tropical to temperate environment that occurred after the initial diversification period of the Clupeoidei. These results combined with our ancestral range reconstruction hypothesis suggest that the probable region of origin and diversification of the Clupeoidei during the Cretaceous period was the tropical marine precursor to the present Indo-West Pacific region. Thus, our study favors the hypotheses of “Region of origin” and “Tropical conservatism” to explain the origins of the longitudinal and latitudinal gradients of clupeoid species richness, respectively. Additional geological and paleontological evidence further define the tropical marine paleo-region of origin as the eastern Tethys Sea region. The Cretaceous fossil record of the Clupeoidei is partially incongruent with the results here as it contains taxa found outside this region. We discuss three possible causes of conflict between our biogeographical hypothesis and the distributions of the Cretaceous clupeoid fossils: regional extinction, incomplete taxonomic sampling and incorrect timescale estimation.

## Introduction

Species richness of worldwide-distributed groups of marine organisms is not uniform across regions and general gradients of diversity are apparent. Typically, tropical regions contain more species than temperate regions, a distribution pattern known as the latitudinal gradient in species richness [1]–[3]. Unbalanced species richness amongst the tropical marine regions is also a common pattern of longitudinal gradients of organism diversity [1] with the Indo-West Pacific (IWP) region comprising more species than any other tropical region [4], [5]. These longitudinal and latitudinal gradients increased dramatically in strength through the Cenozoic era to reach their present maximum [1]. Two prevalent hypotheses have been proposed to explain the formation of heterogeneous diversity patterns along the longitudinal gradient [6]–[8]. The “Region of origin” hypothesis postulates that a widely distributed group of organisms originated and diversified within the region that harbors the maximum species diversity [7]. Then, other regions were colonized through subsequent outward dispersal events. The “Region of accumulation” hypothesis assumes that the region exhibiting the maximum species diversity accumulated species through multiple independent inward dispersal events from peripheral regions [9]. One recent hypothesis has been proposed to explain the formation of heterogeneous diversity patterns along the latitudinal gradient [10], [11]. The “Tropical conservatism” hypothesis holds that a latitudinally widely distributed group of organisms originated and diversified within tropical regions and have spread to temperate regions only more recently [11].

**Figure 1:**
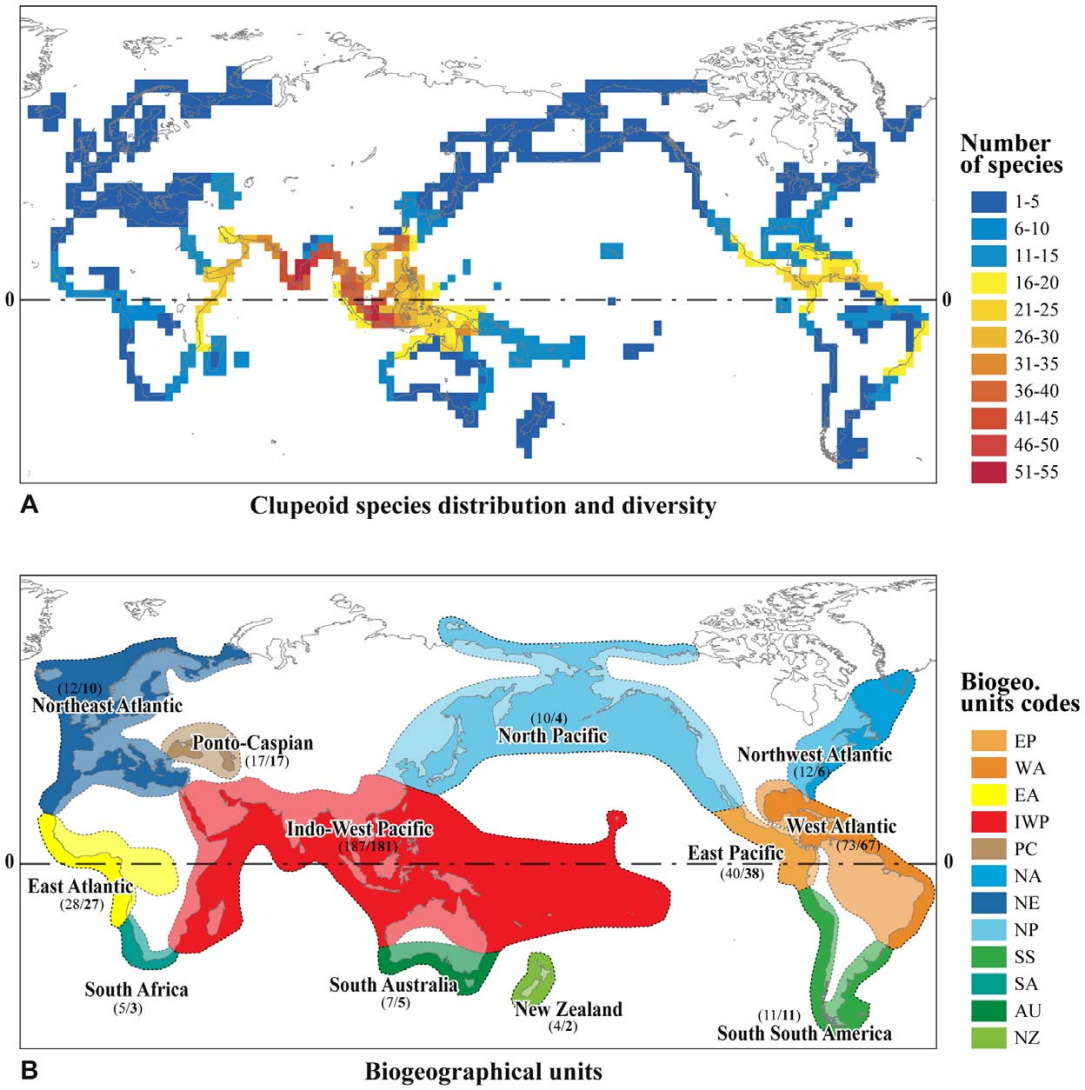
Geographical pattern of distribution of the Clupeoidei. A) Distribution and species diversity of Clupeoidei. Number of species per grid cell (4 by 4 degree latitude-longitude resolution) is represented by cool (low diversity) to warm (high diversity) colors. Individual species distribution compiled from Whitehead [16] and Whitehead et al. [17], Wongratana [125], Gourène and Teugels [126], Peng and Zhao [127], Castro-Aguirre and Viverro [128], Randall [129], Nelson and McCarthy [130], Siebert [34], Britz and Kottelat [131], Menezes and De Pinna [132], Stiassny [97], Castro-Aguirre et al. [133], Roberts [134], Borsa et al. [135], Kimura et al. [136], Loeb [13], DiBattista et al. [14] and Randall and DiBattista [137]. B) The 12 biogeographical units used in the ancestral ranges reconstruction analysis; each unit was delimited by landmasses, vast expanses of open ocean and water temperature. Within each region, total number of clupeoid species (in regular) and number of endemics (in bold) are indicated in parentheses.

Sardines, herrings, anchovies and their relatives (Clupeoidei) include 397 extant species classified into five families: the Clupeidae (55 genera, 205 species), Engraulidae (17 genera, 145 species), Pristigasteridae (9 genera, 38 species), Sundasalangidae (one genus, 7 species) and Chirocentridae (one genus, 2 species) [12]–[15]. They exhibit a complex pattern of worldwide distribution (Fig. 1A), with marine, euryhaline and freshwater representatives as well as tropical, temperate and sub-Artic representatives [16], [17]. Clupeoid species richness amongst regions follows the two general longitudinal and latitudinal gradients of diversity. The majority–about 250 species–of the marine (and euryhaline) species of Clupeoidei lives in near-shore, tropical environments. These marine tropical species reach their highest diversity in the IWP region with more than 180 species (Fig. 1A and 1B). About 60 species of Clupeidae and Engraulidae live in temperate marine waters of both hemispheres (between the isotherms 10°C and 25°C). Finally, about 90 species of Clupeidae, Engraulidae, Pristigasteridae and Sundasalangidae are predominantly freshwater and are found in all continents except Antarctica.

Most of the species and a large majority of genera of the Clupeoidei have a restricted geographical distribution, confined to only one of the 12 defined regions shown in Fig. 1B, suggesting that these fishes have low dispersal capacities. This pattern of endemism is accentuated in tropical regions versus temperate regions. Besides limits imposed by land, two other important factors seem to strongly constrain the inter-regional distribution of clupeoid species: vast expanses of open ocean and water temperature. The historical role of a potential third barrier, salinity, is apparently more complex as within many independent lineages and at various taxonomic levels (from family to species/population), marine, euryhaline and freshwater taxa coexist [16], [17].

Despite its broad and complex pattern of distribution and the supposed low dispersal capacities of its members, there is still no general biogeographical hypothesis to explain the distribution of this group. This is mainly because of the difficulty in determining the phylogenetic intra-relationships of the Clupeoidei and, in particular, of the speciose family Clupeidae [16], [18], [19]. Indeed, only the biogeography of some closely related species groups of Clupeoidei have been examined [20]–[23].

Herein, we offer the first biogeographic hypothesis for the main lineages of Clupeoidei based on a large mitogenomic dataset. We use a Bayesian method to simultaneously reconstruct the phylogenetic relationships and divergence times, and likelihood methods to reconstruct ancestral habitat preferences and ancestral area distributions. Specifically, we want to test if the “Tropical conservatism” hypothesis explains the latitudinal gradient of clupeoid species richness and if the tropical Indo-West Pacific region is a region of origin or of accumulation for clupeoids.

## Results

### Time-Calibrated Phylogenetic Results

**Figure 2:**
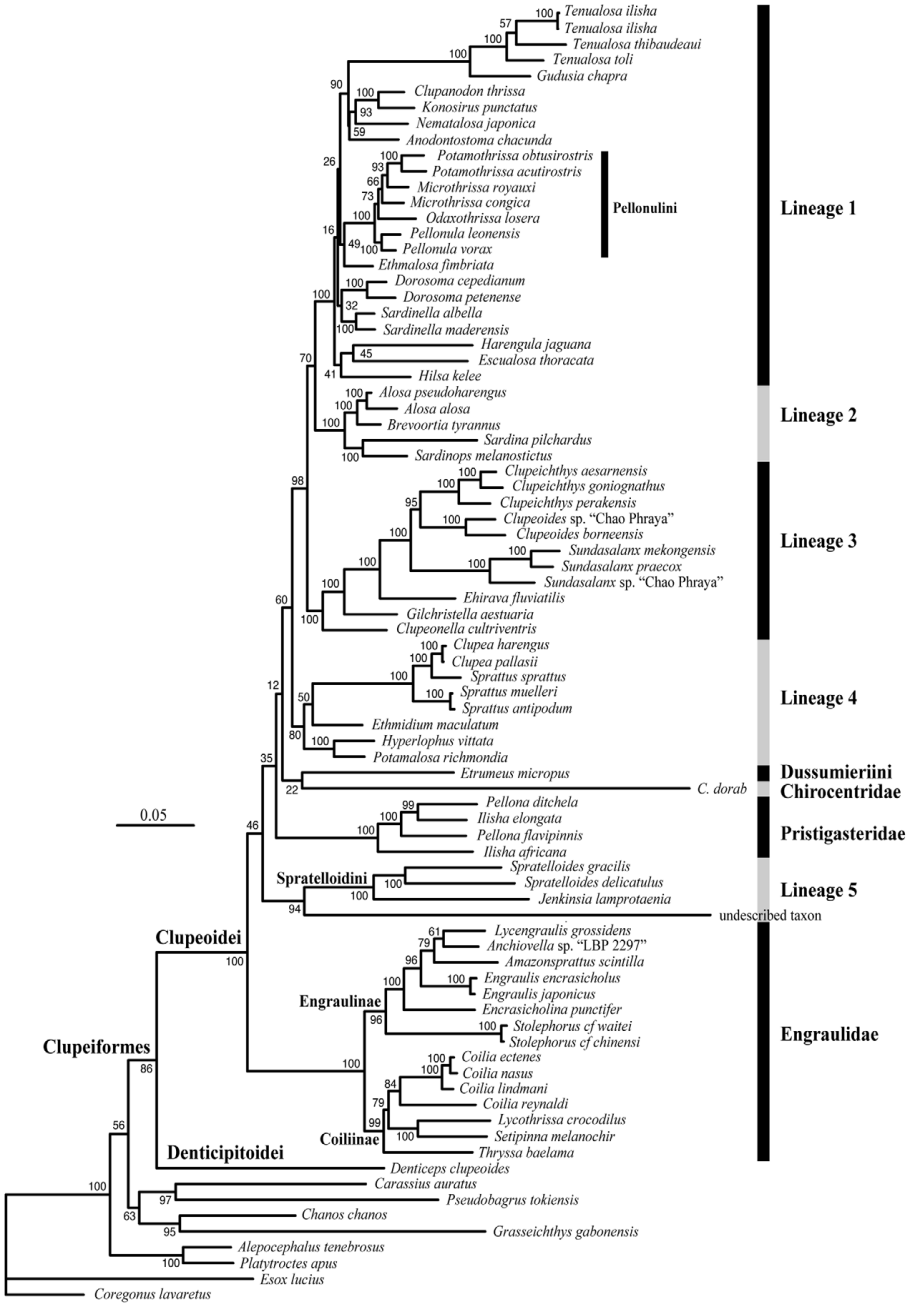
Maximum likelihood tree of the Clupeoidei from analysis of the mitogenomic dataset (using RAxML [99]). Branch lengths are proportional to the number of substitutions per nucleotide position (scale bar = 0.05 substitutions). Numbers at nodes are Bootstrap proportions (in percentage). The tree is rooted with *Coregonus lavaretus* and *Esox lucius*. Abbreviation: *C.*, *Chirocentrus*.

**Figure 3:**
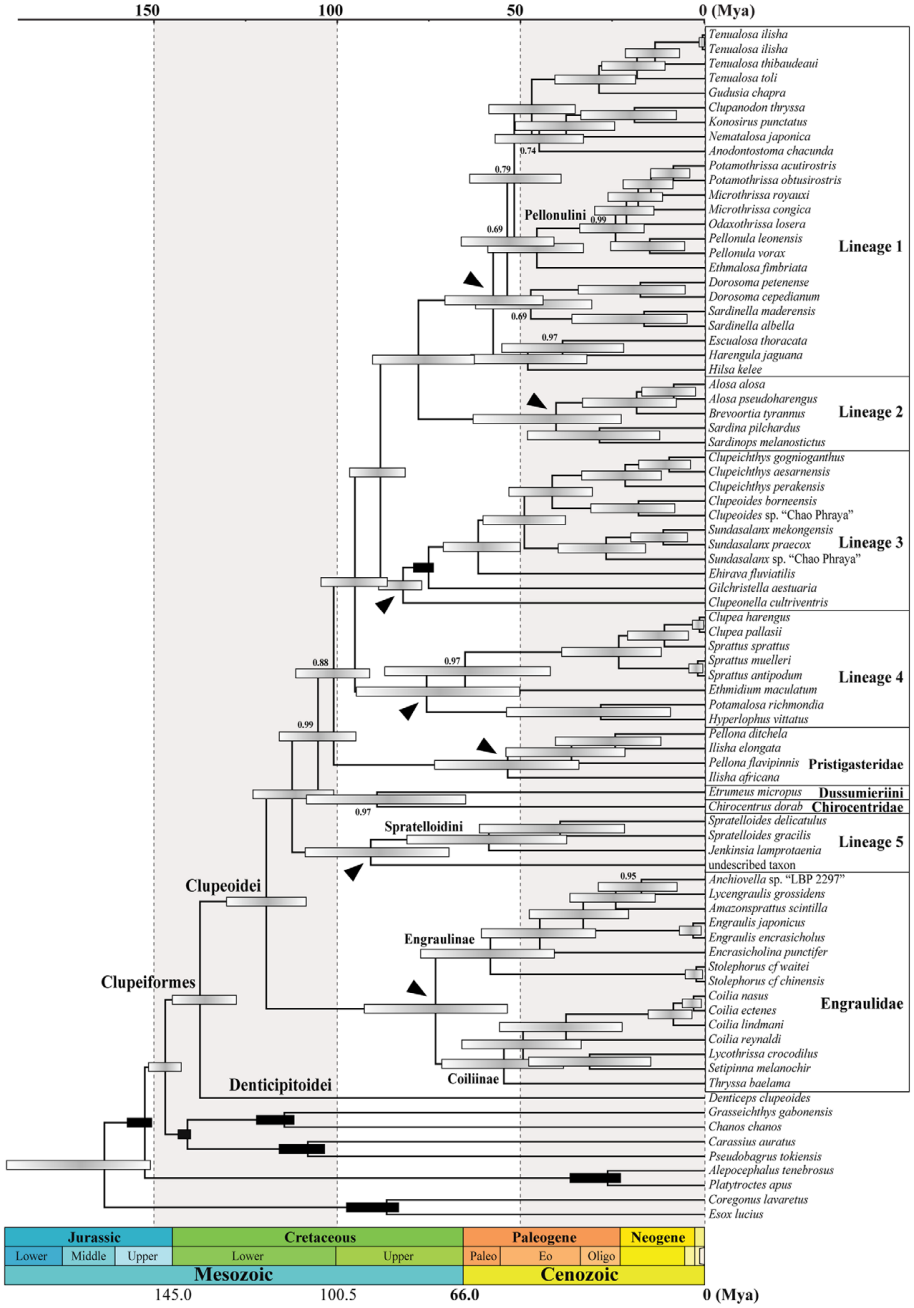
Phylogenetic chronogram of the Clupeoidei based on a Bayesian relaxed clock analysis (using BEAST v1.7.4 [103]) of the mitogenomic dataset, calibrated with seven fossil-based constraints (see text for details). *Coregonus lavaretus* and *Esox lucius* are together used to root the tree. Horizontal timescale is in million years before present (Mya) (Paleogene Epoch abbreviations: Paleo, Paleocene; Eo, Eocene; and Oligo, Oligocene). Black horizontal bars (indicating calibration constraints on the corresponding nodes) and light grey gradient horizontal bars at nodes are 95% age credibility intervals. Numbers given at nodes are the Bayesian posterior probabilities when <1. Black arrowheads indicate the crown group origins of lineages of Clupeoidei as discussed in the text.

Maximum-likelihood and Bayesian analyses recovered nine well-supported monophyletic higher-level groups within the Clupeoidei (Fig. 2, 3 and S1) in agreement with previous molecular studies [24]–[28]. The origin of each of these lineages either pre-dates or is contemporary to the Cretaceous/Paleogene boundary (hereafter designed as the K-Pg boundary) marked by the fifth and last mass extinction. Only three of these nine lineages correspond to currently recognized family groups [18]: the Engraulidae, Pristigasteridae and Chirocentridae. Five other lineages are of mixed content (informally identified as Lineages 1 to 5 in Fig. 2, 3 and S1). The last lineage corresponds to the genus *Etrumeus*. The family Clupeidae and none of its five subfamilies are monophyletic. The paedomorphic genus *Sundasalanx* (Sundasalangidae) is deeply nested within Lineage 3, as the sister group of the Southeast Asian freshwater genera *Clupeoides* and *Clupeichthys*. Together, Lineages 1, 2 and 3 form a well-supported monophyletic group. The relationships among the other lineages are only moderately supported.

### Habitat Preference Evolution

**Figure 4:**
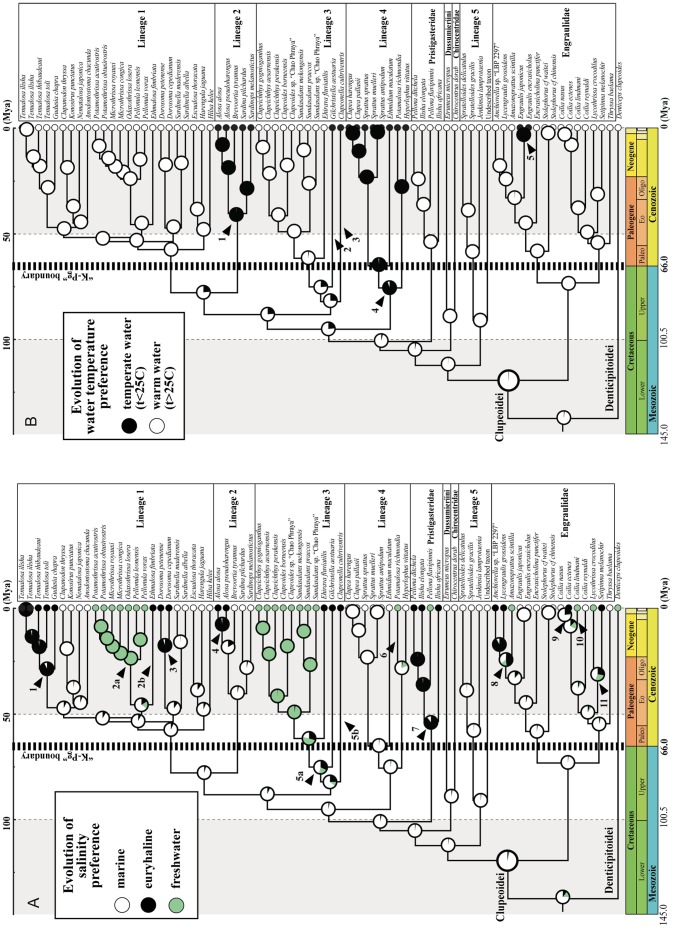
Reconstructions of the evolution of salinity and water temperature preference within the Clupeoidei using likelihood optimization on the Bayesian time-tree topology (see Fig. 3). A) Salinity preference classified in three states: “marine” indicated in white, “euryhaline” in black, and “freshwater” in green. At each node, the relative probabilities of each state (sum = 1) are drawn using pie charts. Black arrowheads indicate transition from marine to euryhaline or freshwater environments. B) Water temperature preference classified in two states: “tropical” indicated in white and “temperate” in black. At each node, the relative probabilities of each state (sum = 1) are drawn using pie charts. Black arrowheads indicate transition from tropical to temperate environments.

The character evolution reconstructions of the salinity and temperature preferences indicate that the most recent common ancestor of the Clupeoidei was very likely marine and tropical (Fig. 4A and 4B). We inferred a minimum of 11 transitions from marine to euryhaline or freshwater environments within six of our nine main lineages; most of these transitions occurred after the K-Pg boundary (Fig. 4A). We inferred a minimum of five transitions from a tropical to temperate environment within four of our nine lineages. Only one of these transitions predated the end of the Cretaceous period and three others may have predated it but their precise dating is difficult to determine because of the internal long branches supporting these temperate taxa (Fig. 4B).

### Ancestral Area Reconstruction

**Figure 5:**
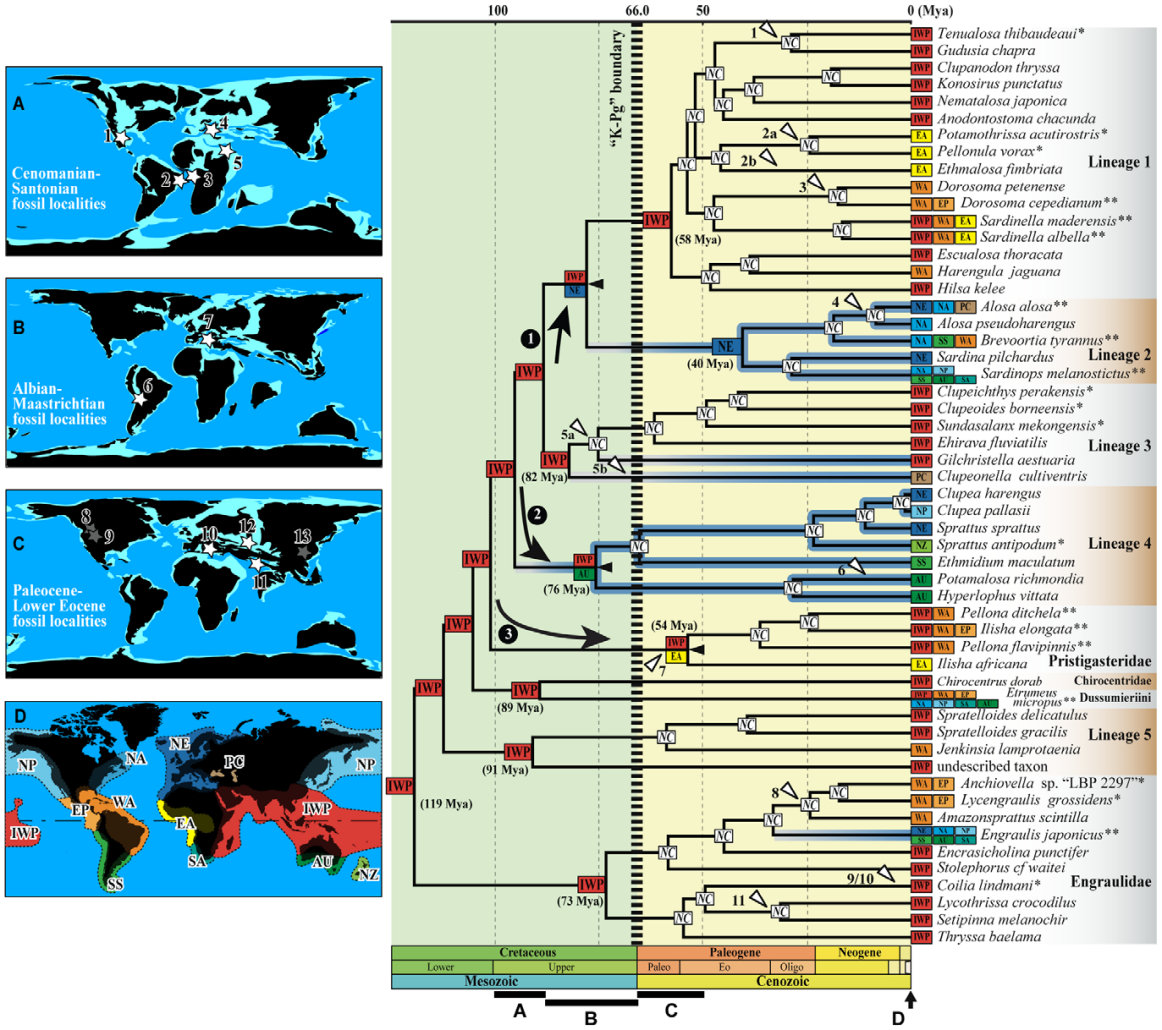
Most likely ancestral ranges reconstruction of the Clupeoidei during the Cretaceous and early Cenozoic period using the dispersal–extinction–cladogenesis (DEC) model [116], [117] onto a simplified Bayesian phylogenetic chronogram. Outgroups (i.e., non-clupeoids) were deleted and biogeographically redundant clupeoid taxa were merged with their respective sister group (see material and methods for details). Ancestral ranges at nodes within each major lineage not reconstructed. Horizontal timescale in million of years ago (Mya) (Paleogene epoch abbreviations: Paleo, Paleocene; Eo, Eocene; and Oligo, Oligocene). Most likely ancestral ranges reconstruction at nodes indicated by code-color boxes (see Fig. 1B for correspondence between regions and two or three-letter codes and colors). Black arrows indicate the three dispersal events predating or likely predating the K-Pg boundary and black arrowheads indicate subsequent allopatric cladogenesis. Temperate lineage branches are underlined in blue and white arrowheads indicate marine to freshwater transitions. “*” after a species name indicates that closely related species to this species have been pruned (see material and methods for details); “**” after a species name indicates that this species is a representative of a supra-specific group having a larger geographical distribution. “*NC*” at nodes indicate that the ancestral ranges were not estimated at these nodes. On the left side, the spatio-temporal context is illustrated with four schematic paleoreconstructions (at 90, 65 and 50 Mya) on which are indicated the temporally corresponding clupeoid fossil localities by white (marine/brackish) and grey (freshwater) stars. Emerged lands are displayed in black and marine environments in blue with the shallow parts in lighter blue. One additional reconstruction (D) shows the current geographical context with the biogeographical units. The clupeoid fossil localities are: 1- the Cenomanian locality “Loma la Mula” in Coahuila, northeastern Mexico (taxon:†*Scombroclupea occidentalis* currently considered as a clupeid *incertae sedis*) [88]; 2- the marine shale yielded in the Taquari Member (Albian) of Riachuelo Formation (state of Sergipe, Northeastern Brazil) (taxon:†*Nolfia riachuelensis* currently considered as a clupeid *ad interim*) [81]; 3- the marine deposit from the Cenomanian of Kipala, Democratic Republic of Congo (taxon:†*Nolfia kwangoensis* currently considered as a clupeid *incertae sedis*) and the marine Santonian of Vonso, Democratic Republic of Congo (taxon:†*Audenaerdia casieri* currently considered as a clupeid *incertae sedis*) [85], [89]; 4- the Cenomanian (Upper Cretaceous) Komen (Slovenia) fossil lagerstätte (taxon: †*Scombroclupea macrophthalma* currently considered as a clupeoid *incertae sedis*); [83], [138]; 5- the Cenomanian fossil fish localities of Lebanon (e.g., Namoura, Hakel and Hajula) (taxa: †*Scombroclupea* spp. currently considered as clupeoids *incertae sedis*) [83]; 6- the Upper Cretaceous (Maastrichtian) of Cayara, El Molino Formation, Bolivia (taxon:†*Gastroclupea branisai* currently considered as a pristigasterid *incertae sedis*) [39], [81]; 7- the Upper Cretaceous (Campano-Maastrichtian limit, 74.0 Mya) marine sediments of Nardò, Italy (taxa: †*Portoselvaggioclupea whiteheadi* and †*Nardoclupea grandei* [Dussumieriinae], †*Pugliaclupea nolardi* [Clupeinae], †*Lecceclupea ehiravaensis* [Pellonulinae], and †*Italoclupea nolfi* [Alosinae]) [51]–[54]; 8- The Middle Paleocene Tongue River Formation (lacustrine limestone), near Bay Horse, Montana, USA (taxon: †*Knightia vetusta* currently considered as a clupeoid *incertae sedis*) [40]; 9- the Middle Eocene Laney Member of the Green River Formation, southwestern Wyoming, USA (lacustrine deposits)(taxon: †*Gosiutichthys parvus* currently considered as a clupeoid *incertae sedis*) and the Lower Eocene lacustrine sediments of Wyoming, Colorado and Utah, USA (taxa: †*Knightia alta* and †*Knightia eocaena* currently considered as clupeoids *incertae sedis*) [40]; 10- the Lower Eocene (52.0 Mya) marine sediments of Monte Bolca, Italy (taxa: several species of Clupeidae including at least one species of Dussumieriinae *sensu* Grande [18]); 11- the Upper Paleocene freshwater lacustrine deposits of Bamanbor and Ninania of Saurashtra, India (taxon: †*Horaclupea intertrappea* currently considered as a clupeid *incertae sedis*) and the Eocene Saline Series of the Salt range of Pakistan (taxon: the clupeid †*Horaclupea geei*) [18]; 12- the Late Paleocene fish fauna of the Danata Formation in Turkmenistan (taxon: †*Primisardinella genetrix* currently considered as a clupeid *incertae sedis*) [18] and 13- the Eocene (probably freshwater) deposits of Hupei, China (taxon: †*Knightia yuyanga* currently considered as a clupeid *incertae sedis*) [18].

The ancestral area reconstruction analysis using the DEC (*Dispersal-Extinction-Cladogenesis*) model on our simplified time-calibrated phylogeny produced a most likely hypothesis for the distribution of the most recent common ancestor and the region of early diversification of the Clupeoidei (Fig. 5). The most significant results can be summarized as follows: 1- The most recent common ancestor of the Clupeoidei lived in the proto-IWP region at about 119 Mya (million years ago; Lower Cretaceous); 2- during the rest of the Cretaceous, the diversification of Clupeoidei predominantly necessitated within-IWP events of speciation; 3- six of the nine main lineages originated within the IWP region; 4- at the end of the Cretaceous, two, likely three, dispersal events occurred. The first event of dispersion from the IWP was northward, to the Northeastern Atlantic (leading to the most recent common ancestor of Lineages 1 and 2); the second event of dispersion from the IWP was southward, to south Australia (leading to the most recent common ancestor of Lineage 4); the likely third event of dispersion from the IWP was towards the East Atlantic (leading to the most recent common ancestor of the Pristigasteridae). The first two events may have been of similar ages and they were associated with tropical-to-temperate habitat transitions. The timing of the third event is difficult to estimate because of our incomplete taxonomic sampling within the Pristigasteridae and the long internal branch supporting our crown group Pristigasteridae. Each expansion event was followed by a diversification event between the IWP and the newly colonized region.

## Discussion

### Phylogenetic Relationships

Our taxonomic sampling was designed to investigate the higher-level relationships and early biogeography of the Clupeoidei, and significantly improved upon earlier molecular studies with the addition of 32 new taxa. The phylogenetic results are consistent with previous molecular studies in recovering the same major lineages [24]–[29]. Three of these lineages are the families Pristigasteridae, Chirocentridae and Engraulidae; each of these families is well defined by several morphological synapomorphies. A fourth lineage is made up by the genus *Etrumeus* (Dussumieriini). Five lineages are each composed of species previously classified in different clupeid subfamilies (informally identified as Lineages 1 to 5 in Fig. 2 and 3), making all traditional clupeid subfamilies non-monophyletic. None of these five lineages were previously formally recognized using morphological characters.

Lineage 1 is a large circumtropical clade that contains the dorosomatin *Dorosoma*, *Nematalosa*, *Clupanodon*, *Konosirus* and *Anodontostoma* [30] along with several taxa previously classified within the Clupeinae (e.g. *Harengula* and *Escualosa*), Alosinae (e.g. *Tenualosa* and *Gudusia*) and Pellonulinae (African freshwater tribe Pellonulini). Nelson [19], [31] observed that the gill-rakers of several of these taxa do not overlap; therefore this character may represent a synapomorphy of this group.

Lineage 2 is restricted to four predominantly temperate genera, *Alosa*, *Brevoortia*, *Sardinops* and *Sardina*. Nelson [19], [31] noted the close relationship between *Sardinops* and *Sardina*, and the possible relationship of these two genera with *Alosa*. Neither Nelson [19], [31] nor Grande [32] considered *Brevoortia* as closely related to *Alosa*, *Sardina* and *Sardinops*.

Roberts [33] described the family Sundasalangidae to classify a new diminutive genus, *Sundasalanx*, that he considered closely related to the family Salangidae (Osmeriformes). Siebert [34] proposed that *Sundasalanx* is indeed a clupeid possibly related to the genus *Jenkinsia*. Ishiguro et al. [35] confirmed the placement of *Sundasalanx* within the Clupeiformes, but as a distinct family (Sundasalangidae) of uncertain affinities. Subsequent molecular studies did not provide better evidence to resolve the phylogenetic position of *Sundasalanx* within the Clupeiformes because of incomplete taxonomic sampling [24], [26], [27], [36]. In the present study, *Sundasalanx* is deeply nested within Lineage 3, and its phylogenetic position is resolved as the sister group of the clade (*Clupeichthys*, *Clupeoides*).

Lineage 4 includes the economically important temperate genera *Clupea* and *Sprattus*, along with *Ethmidium* (previously classified within the Alosinae), and *Potamalosa* and *Hyperlophus* (previously classified within the Pellonulinae). *Ethmidium*, *Potamalosa* and *Hyperlophus* are so-called “doubled-armored herrings” in having a complete series of ventral and dorsal scutes, a rare character within the living Clupeoidei; incomplete series of dorsal scutes occurs in *Clupanodon* [37], *Pristigaster* and some other clupeid and engraulid taxa [38], [39]. The “doubled-armored herring” genera were not considered closely related to each other. Our finding shows that *Ethmidium*, *Potamalosa* and *Hyperlophus* belong to the same lineage, even if they do not form a monophyletic group. *Ethmidium* is more closely related to the clade (*Clupea*, *Sprattus*) than to either *Potamalosa* or *Hyperlophus*. This suggests the possible single evolutionary origin of their complete series of dorsal scutes, secondarily lost in the clade (*Clupea*, *Sprattus*). This result may have implications for reconsidering the phylogenetic position of “doubled-armored herring” fossils such as †*Knightia* [40].

While recent studies recognize the same major lineages within the Clupeoidei, the relationships among them are still unsatisfactory resolved. Currently, molecular data support the monophyly of Lineages 1, 2 and 3 together with substantial statistical support (Fig. 2 and 3) [24], [26]–[28], [36]. Lineage 5, along with *Chirocentrus* and *Etrumeus*, do not form a monophyletic group, contrary to what Lavoué et al. [24] inferred. This incongruence is likely the consequence of the weak phylogenetic signal in this part of the tree. The clarification of the relative positions of these three groups requires additional studies. The mitochondrial genes alone moderately support the hypothesis that Engraulidae is the sister group of the rest of the Clupeoidei [28] (this study) while, so far, the few nuclear genes examined tend to support an alternative hypothesis in which the Pristigasteridae is the sister group of the rest of the Clupeoidei [27], [36]. Miyashita [41] identified a unique derived character shared by the Clupeidae, Pristigasteridae and Chirocentridae, therefore supporting the sister-group relationship between these three families and the Engraulidae (*Sundasalanx* was not examined by Miyashita [41]). Di Dario [42], [43] published morphological evidence that contrast with this hypothesis.

### Region of Origin and Early Diversification of the Clupeoidei

As defined herein, the IWP region stretches longitudinally from the southeasternmost tropical coast of Africa across the southern continental margin of the India-Eurasia landmass into the West Pacific Plate, further east to the tropical South China Sea (Taiwan), the Hawaiian Islands, Micronesia, Polynesia and Northeastern Australia (Fig. 1B) [5], [44]. At the global scale, the IWP forms the largest tropical region and it is celebrated for its exceptional biodiversity including more than 4,000 species of fishes, most of them endemic [5], [45], [46]. This is by far more species than in any other marine (tropical and non-tropical) region of the World.

The regional species richness pattern of the Clupeoidei (Fig. 1A and 1B) parallels those of several tropical coral reef fish groups or other organism groups in reaching its maximum within the central part of the IWP region [5]. This region contains almost half of all known clupeoids (187 species), with more than 95% of the clupeoid species living in the IWP region being endemic (180 species). Our ancestral habitat preference and ancestral area distribution reconstruction suggest that the tropical marine IWP region is not only the region of highest diversity of the Clupeoidei but also their region of origin and early diversification (Fig. 5). Therefore, the “Region of origin” hypothesis along with the “Tropical conservatism” hypothesis may account for the observed longitudinal and latitudinal gradients of Clupeoidei, respectively.

Based on fossil distributions, phylogenetic inferences and biogeographical reconstructions, it has been proposed that the precursor of the IWP region during the Cretaceous/Paleogene period was the eastern Tethys Sea region in which several tropical marine IWP taxa originated [4], [47]–[50]. In this regard, two fossil localities in Italy are of particular interest to document the early evolution of tropical fish community of the eastern Tethys Sea region during the Upper Cretaceous and the early Cenozoic [4], [51]–[54]. From the first locality, the Upper Cretaceous (Campano-Maastrichtian limit, 74.0 Mya) marine sediments of Nardò, six clupeid species assigned to four different subfamilies have been recently described [51]–[54]. Of the 250 fish fossil species that have been described from the second locality, the Eocene marine sediments of Monte Bolca, there are no less than 12 clupeid-like species [4], [18]. Although the phylogenetic positions of most of these fossils need to be clarified [18], the large number of clupeid-like species at these two localities tends to indicate that the tropical marine clupeoids had already significantly diversified in the eastern Tethys Sea region in the Upper Cretaceous/Early Eocene. This is consistent with our molecule-based hypothesis that the eastern Tethys Sea was the IWP precursor region where the early diversification of the Clupeoidei occurred.

### Evolutionary Habitat Transitions

Evolutionary transitions between marine and freshwater environments and between tropical and temperate environments are perceived as rare events on a macroevolutionary scale [55], [56].

The observation of the environmental preferences of some taxa within several lineages of Clupeoidei suggests these fishes possess inherited physiological capacities to adapt to a large range of salinity conditions [57]–[59]. Thus, the salinity barrier is not perceived as a strong barrier of dispersion and colonization in these fishes. On a macroevolutionary scale, we infer multiple and independent transitions from marine to freshwater environments. All these transitions occurred late in the evolution of Clupeoidei and the early clupeoids were confined to marine habitat, at least, until the end of the Cretaceous Period. Our sampling is incomplete within most of the main lineages and we anticipate that the total number of transitions will be higher than the 11 transitions inferred when additional freshwater taxa are examined (*e.g., Rhinosardinia*, *Pristigaster*, *Gonialosa*, etc.); we also predict, however, that none of the potential additional transitions would occur earlier than the Cenozoic because our taxonomic sampling likely includes representatives of all major clupeoid lineages.

Water temperature preference is different given that it appears to represent a stronger dispersal barrier for the clupeoids. We detected only 5 transitions from tropical to temperate habitats and it is likely that we have already identified all tropical-to-temperate transitions because our taxonomic sampling covers the higher-level diversity of the temperate clupeoids. Interestingly, one, maybe four, of these transitions occurred at the end of the Cretaceous Period or early in the Cenozoic Era, at a time of significant (although erratic) global cooling. Most of the Cretaceous Period was characterized by a warm climate [60] with surface temperatures and sea surface temperatures at low and high latitudes higher than those of today [61]–[65], owing to a lack of permanent ice sheets [65], . At that time, the latitudinal environmental gradient was at its minimum with rather uniform environmental conditions from equatorial regions to pole regions [64] and the latitudinal taxonomic gradient was correspondingly reduced [1]. Indeed, the cooling of the average global temperature at the end of the Cretaceous Period [67] may have resulted in new opportunities for temperature-mediated allopatric speciation at higher latitudes for the Clupeoidei.

### Tree Topology Uncertainties and Taxonomic Sampling Quality

Potential issues may lead to incorrect estimations of ancestral habitats and ancestral area distributions [68], [69]. Two important issues in this study could affect the reliability of the biogeographical hypothesis: the topological uncertainties of the phylogenetic tree [69] and the quality of the taxonomic sampling [70], [71].

The higher-level phylogeny of the Clupeoidei is difficult to accurately reconstruct because of an overall lack of signal among the main lineages (Fig. 2 and 3) and several alternative branching orders cannot be rejected [18], [24], [27], [43]. Nylander et al. [69] recommended that biogeographical reconstruction methods should take into account such phylogenetic uncertainties because different tree topologies may lead to different biogeographical conclusions. However, we consider that alternative branching orders among the nine main lineages of Clupeoidei will still establish the IWP as the unique region of origin of this group. This is because six of the nine clupeoid lineages originated within the (tropical marine) IWP region and the last three lineages originated in marine areas formed by two sub-regions, one of which is the IWP (Fig. 5). This yields well-supported ancestral area reconstruction results.

An incomplete, geographically biased taxonomic sampling could also lead to incorrect biogeographical inference [70], [71]. In our study, the quality of the taxonomic sampling within the family Clupeidae is the most critical because: 1- this family is not monophyletic whereas each of the other four families is monophyletic (*e.g.,* Pristigasteridae, Sundasalangidae, Chirocentridae and Engraulidae), and 2- the clupeid taxa contribute to no less than six of the nine major lineages. Therefore, missing clupeid taxa that do not occur within the IWP region may potentially represent additional lineages that could modify the biogeographical scenario. Despite our efforts to broadly cover the taxonomy diversity and distribution of the Clupeidae, 13 genera occurring outside the IWP region are missing.

We argue that the addition of these genera to our dataset will have modest consequences on our main biogeographical conclusions for the following reason: previous morphological and molecular work, along with our phylogenetic framework, allow us to predict the phylogenetic positions of at least 10 of these 13 genera within one of the identified major lineages with high confidence. Six missing West Central African freshwater genera, *Sierrathrissa*, *Nannothrissa*, *Laeviscutella*, *Thrattidion*, *Limnothrissa*, and *Stolothrissa*, are considered to be members of the tribe Pellonulini that is deeply nested within Lineage 1 [16], [28], [72] (Fig. 2 and 3). The phylogenetic position of the seventh missing Central African freshwater genus *Congothrissa* is still controversial as it is either placed in its own family of uncertain affinities [72], [73] or it is classified within the tribe Pellonulini [16], [74]. The two tropical South American genera, *Opisthonema* and *Rhinosardinia*, belong to Lineage 1 [18], [19], [27], [28], [75] and the two temperate South American genera, *Ramnogaster* and *Strangomera*, are very likely nested within Lwineage 4 [16], [76], [77]. Finally, the phylogenetic positions of only two South American clupeid genera, *Lile* and, in particular, *Platanichthys* are uncertain because there is little comparative evidence relative to these two genera. Whitehead [78] who erected the genus *Platanichthys*, stated that this genus “*which combines features of both the Clupeinae and the Pellonulinae, raises many problems which cannot be solved without a complete review of the Clupeidae*”.

### Paleodistribution of Cretaceous Clupeoids

The fossil record is the only direct evidence of the past and provides important information on ages, palaeodistributions and paleoenvironments of the organisms. Occasionally the biogeographical hypotheses inferred from the distribution of extant taxa are incongruent with palaeodistributions because of a number of natural and artifactual causes [79], [80]. Our ancestral area reconstruction using the distribution of the extant Clupeoidei is unequivocal in identifying the IWP as the region of origin and early diversification of the Clupeoidei during the Cretaceous Period. However, there is paleontological counterevidence that the precursor of the IWP region (*i.e.,* the eastern Tethys Sea region) was the unique region of origin and early diversification of the Clupeoidei, during the Cretaceous Period. Several Cretaceous clupeoid fossils have been described [18], [51]–[54], [81]–[85] and their distributions challenge our biogeographical hypothesis as they have been found not only in the eastern Tethys Sea region [83] but also in different marine tropical regions, including the emerging West and East tropical Atlantic regions, soon after the early separation of Africa and South America [81], [82], [84] (Fig. 1B and 5). It should be noted, however, that the Tethys Sea was widely connected to several marine regions. For example, the Tethys Sea and the South Atlantic Ocean were intermittently connected through North-South Trans-Saharan seaways [86]–[87]. This may explain the distributions of the fossils (of Cretaceous age) found in West Africa and Northeast Brazil (Fig. 5).

The reason for such incongruence is difficult to establish because the precise phylogenetic positions of these Cretaceous clupeoids relative to the extant clupeoid taxa are mostly unresolved. The Cretaceous clupeoid taxonomy and systematics lack a comprehensive revision. The Santonian (83.5–85.8 MYA) brackish †*Gasteroclupea branisai* of Bolivia is currently identified as a stem pristigasterid, but additional investigation of this fossil is desirable [18]. Forey et al. [83] left *incertae sedis* within the Clupeoidei the position of the Cenomanian genus †*Scombroclupea* whereas Gierch et al. [88] considered †*Scombroclupea occidentalis* as a clupeid. De Figueiredo [81] admitted that there is little morphological evidence to place the most ancient (of Albian age, 99.6–112 Mya) crown group clupeoid fossil †*Nolfia riachuelensis* and the Cenomanian †*Nolfia kwangoensis* [85] within the Clupeidae (placed *ad interim*). The Santonian †*Audenaerdia casieri* (West Central Africa) may represent an early clupeid (*incertae sedis*) [89] that could be classified either in the subfamily Clupeinae or Alosinae [84], two non-monophyletic groups in our study.

Taking into account the uncertainties in the phylogenetic positions of these fossils, we identify and discuss three possible causes that could explain the difference between our biogeographic reconstruction and the paleodistributions of the Cretaceous fossils: 1- regional extinction, 2- incomplete taxonomic sampling and 3- inaccurate molecular timescale.

Supposing that the Cretaceous fossils represent early lineages within the crown group Clupeoidei, these lineages may have gone extinct through regional selective extinction events. Different rates of extinction among regions can strongly obscure the biogeographical pattern and process of extant organisms [79], [80], [90]. The effects of extinction are more pronounced for older taxa that may have faced more extinction events. For example, the early Cretaceous clupeoids faced the K-Pg mass extinction episode. On the other hand, the living sister group(s) of these Cretaceous fossils may have not yet be identified because of the incompleteness of our taxonomic sampling. As discussed in the previous section 4, the missing South American *Platanichthys* and *Lile* or the African *Congothrissa* could hypothetically represent distinct and old lineages within the Clupeoidei, sister group to Cretaceous clupeoids.

Supposing that the Cretaceous fossils belong to the crown group Clupeoidei and they are nested within some of our inferred main lineages of Cenozoic age, it will demonstrate that the inferred time divergences of these lineages are underestimated. In molecular dating reconstruction, selected fossils used to calibrate the clock provide minimum ages of the clades in which they belong to (*i.e.,* a clade can’t be younger that its older member). The use of fossil-based maximum age (soft or hard) constraints, as we did (see material and methods section), is more contentious as it relies on the perception of the quality of the fossil record: the oldest fossil of a group having a rich fossil record provides a minimum age for the origin of this group but also an estimation of its maximum age. Several recent molecular studies provided timescale estimations for the diversification of the Teleostei using either minimum and maximum age constraints [28], [84], [92] or only minimum age constraints [93]–[96]. These latter studies estimated the age of the crown group Clupeoidei (i.e., the time divergence Engraulidae/Clupeidae) to about 150 MYA. This is more than 30 MY older than the mean age (119 MYA) of the most recent common ancestor of the Clupeoidei we inferred and more than 40 MY older than the age of the first clupeoid fossil [81]. At present, we have no means of deciding which of these divergence time reconstructions provide the best estimate for the absolute age of the Clupeoidei but we acknowledge the possibility that the Clupeoidei diversification may be older than the timescale we have inferred here.

### Additional Biogeographical Patterns

Although our taxonomic sampling was not designed to further investigate the biogeography within each of the nine inferred lineages, we observe that the distribution patterns of some lineages deserve further attention. For example, Lineages 2 and 4 contain only temperate taxa that were previously considered unrelated whereas Lineage 1 includes only tropical taxa. Lineage 4 exhibits a bipolar distribution, with the northern clade (*Sprattus sprattus*, *Clupea* spp.) nested within the southern one (including *Sprattus muelleri*, *Sprattus antipodum*, *Ethmidium*, *Potamalosa* and *Hyperlophus*). Within Lineage 3, the Ponto-Caspian *Clupeonella cultriventris* is not closely related to the Atlantic taxa, but instead, it is the sister group of a clade of freshwater and euryhaline species endemic to the IWP region. Examination of *Spratellomorpha* and *Sauvagella* will be critical to reconstruct the biogeography of this lineage [97].

## Materials and Methods

### Ethics Statement

The research was performed at the University of Tokyo in accordance with the University of Tokyo’s guidelines regarding animal research. No ethics statement was required for this project as no experiment involved live fishes. Most of the specimens examined in this study were purchased from local fish markets or directly from professional fishermen. The taxonomic sampling was completed through legal tissue donations from International Research Institutes (see “acknowledgements” section).

### Taxonomic Sampling

We examined 73 clupeoid taxa sampled from all five families, paying special attention to the most speciose but poorly defined family Clupeidae with 49 taxa from 33 genera [16]. As a reference, we used the classification of Clupeoidei of Whitehead [16] and Whitehead et al. [17] with the addition of the family Sundasalangidae as proposed by Siebert [34] and corroborated by Ishiguro et al. [35]. The outgroup selection includes *Denticeps clupeoides* (the sister group of Clupeoidei), two alepocephaliforms, four ostariophyses and two euteleosts. The Japanese population of *Etrumeus teres* from which we have previously determined the complete mitogenome (*mt*) of a specimen (DDBJ/EMBL/GenBank accession number: AP009139) [26] is now considered to be *Etrumeus micropus* [14]. Accordingly, we have revised the species name of this *mt* sequence as *Etrumeus micropus*.

### Mitogenomic DNA Sequencing

Total genomic DNA was extracted from muscle or fin tissue. Whole mitogenomes were first amplified using a long PCR technique and a set of fish-versatile primers [98]. The long PCR products were then use as templates for nested PCR to amplify short and overlapping segments of the entire mitogenomes. Double-stranded DNA products were used as templates for direct cycle sequencing reactions with dye-labeled terminators and labeled fragments were read on a Model 3130 *xl* DNA automated sequencer (Applied Biosystems). The 30 complete or nearly complete *mt* sequences plus the partial *mt* sequences of *Anodontostoma chacunda* (13,848 bp) and *Hilsa kelee* (14,850 bp) were deposited in the DDBJ/EMBL/GenBank database; their accession numbers are given in the Table S1.

### Phylogenetic Analysis and Divergence Time Estimation

Mitogenomic sequence editing and individual gene alignment were performed as previously described [98]. Our data matrix includes 82 taxa and 10,733 positions after excluding the homoplastic third codon positions. Phylogenetic analyses were performed using the partitioned maximum-likelihood (ML) method as implemented in the RAxML-HPC [99] with its graphical interface raxmlGUI 0.93 [100]. We set three partitions according to the first and second codon positions and non-protein coding genes and heuristic searches were conducted under mix models of sequence evolution, which allow individual model parameters of nucleotide substitutions to be estimated independently for each partition in the analysis. As recommended in the manual of RAxML-HPC [99], a GTR+Γ model (with 4 discrete rate categories) for each partition was used without incorporating proportion of invariant sites. Thorough ML tree search was conducted through 100 distinct runs. The optimal tree was determined by comparison of the likelihood scores among the suboptimal trees obtained per run. To evaluate the robustness of the internal branches of the ML tree, 500 bootstrap replications (BS) were calculated under the GTR+Γ model.

A partitioned Bayesian phylogenetic analysis was then conducted with MrBayes 3.1.2 [101], [102]. We set up the same three partitions as described above and selected a GTR+Γ model of sequence evolution. Two independent Bayesian runs were performed, each of 3*10^6^ generations. The 50% majority-rule consensus tree along with the posterior Bayesian probabilities at nodes were calculated from the 54,000 trees pooled from the two runs, after removing the burn-in parts ( = 10%).

Finally, phylogenetic relationships were simultaneously estimated with divergence times and their 95% highest posterior density intervals using a partitioned Bayesian method that incorporated a relaxed molecular clock method and a set of seven fossil-based calibrations (see below), using BEAST v.1.7.4 [103]. We set three partitions according to the first and second codon positions and non-protein coding genes and GTR+Γ models of sequence evolution were assigned for each partition; substitution rate parameters, rate heterogeneity model and base frequencies were unlinked across partitions. Three independent runs of 1×10^8^ generations each were performed using BEAST. Each run was initiated from a user-starting time tree that we built in advance with BEAST using a simple HKY model of sequence evolution, no partition, a strict molecular clock and a single prior age constraint for the root of the tree at 180 millions years ago (Mya). Trees and divergence time estimates were sampled once every 5,000 generations, and each run’s parameters were checked for convergence with the software Tracer v1.5. We removed the burn-in parts of each run ( = 10%) and the remaining tree samples from the three runs were then pooled into a combined file, and the maximum clade credibility tree with mean divergence times and their 95% credibility intervals assigned to the nodes was built using TreeAnnotator v.1.7.4.

Prior age distributions of seven selected nodes follow a exponential distribution with a minimum age equal to the minimum age of the geological stage from which the fossil was excavated and a maximum age (in the 95% credibility interval) equal to the maximum age of the stage. The international stratigraphy chart of the 2012 International Commission on Stratigraphy [available online at http://www.stratigraphy.org/] provides revised ages of the units of time. Our method of calibration is conservative because we assume that the fossil records of some teleost groups are coherent, providing overall good estimates of the diversification through time of these groups. Therefore, selected fossils from these groups provide estimates of minimum and maximum ages for their origins [104].

Clupeiform and clupeoid fossils are abundant [18], [81], [82], [84], [85]. However, Grande [18] who reviewed and commented on the identities of most of these fossils (*N* >150), concluded that the phylogenetic positions of most of them of Cretaceous or early Cenozoic age are equivocal in the absence of a comprehensive synapomorphy-based phylogenetic tree of the extant Clupeoidei. Grande [18] concluded that these fossils should be considered as *incertae sedis* within either Clupeoidei, Clupeiformes or Clupeomorpha. As a consequence we have decided not to consider any clupeiform fossils to time-calibrate our phylogenetic tree, except one clupeoid recently described by Taverne [54]. This fossil, †*Lecceclupea ehiravaensis*, is demonstrably classifiable within the tribe Ehiravini *sensu* Grande [18], [97]. †*Lecceclupea ehiravaensis* is remarkable as it represents the oldest ehiravin fossil known (74.0 Mya) and it significantly extends the temporal occurrence of this group. Therefore, the age of the clade containing *Gilchristella* and *Clupeichthys* is set to a minimum of 74.0 which corresponds to the age of this fossil [exponential distribution offset = 74.0; mean = 5].

We selected six additional fossils from related fish groups having rich fossil records and commonly used as calibration points in molecular time divergence analyses [91], [70], [72], [104]–[106]. They are listed hereafter with their ages and the mean values of exponential distributions indicated in brackets: 1- The age of the Otocephala (the crown group including Clupeiformes, Alepocephaliformes and Ostariophysi) is set to 152.1 Mya (Kimmeridgian Stage, 152.1–157.3 Mya) [exponential distribution offset = 152.1; mean = 1.6] which corresponds to the age of the oldest ostariophysian fossil known, *†Tischlingerichthys viohli* [104], [107], [108]. The gonorynchiform fossil record extends back to the Early Cretaceous [109] and provides two calibration points: 2- the oldest gonorynchiform fossil is †*Rubiesichthys gregalis* [109] from the Berriasian Stage (139.8–145.0 Mya, Lower Cretaceous). This fossil was used to calibrate the age of the crown group Ostariophysi [exponential distribution offset = 139.8; mean = 1.7]; 3- the oldest fossils assigned to the crown group of the Gonorynchiformes are the chanid †*Chanos leopoldi* and †*Nanaichthys longipinnus* from the Aptian Stage (113.0–125.0 Mya) [109], [110]. We therefore use these two fossils to constrain the age of the most recent common ancestor of the clade (*Chanos chanos*, *Grasseichthys gabonensis*) [exponential distribution offset = 113.0; mean = 4.3]; 4- we used †*Santanichthys diasii* [111], the oldest known characiform fossil, to calibrate the minimum age of the clade (*Pseudobagrus tokiensis*, *Carassius auratus*) to 100.5 Mya [exponential distribution offset = 100.5; mean = 5]; 5- the oldest alepocephaliform fossil is the alepocephalid †*Carpathichthys polonicus* of Miocene age [112], which provided us with a minimum age of 23.0 Mya for the clade (*Alepocephalus*, *Platytroctes*) [exponential distribution offset = 23.0; mean = 5]; 6-†*Estesesox foxi* [113] from the Early Campanian stage provided a minimum age of the clade (*Esox*, *Coregonus*) [exponential distribution offset = 83.6; mean = 5].

### Character Evolution Reconstruction

The ancestral habitat preference relative to water temperature and salinity was reconstructed on the maximum clade credibility tree using the “Mk1” evolutionary model as implemented in Mesquite v2.72 [114]. Three character states were assigned for salinity preference: freshwater, euryhaline, and marine. Two character states were assigned for water temperature preference: warm water [*i.e.* tropical, t>25°C] and temperate water [*i.e.* t<25°C]. The few warm-temperate taxa, such as *Etrumeus* spp, were considered as tropical. Temperature and salinity preference estimations for each species were mainly collected from the two volumes FAO species catalogue of “*Clupeoid fishes of the World*” [16], [17] with additional information from Milton [115].

### Ancestral Area Reconstruction

To reconstruct the ancestral areas of the Clupeoidei, we used the DEC (*Dispersal-Extinction-Cladogenesis*) model-based likelihood method using the software Lagrange (*likelihood analysis of geographic range evolution*) version 20100721 [116], [117]. The DEC model relies on a continuous-time model for geographic range evolution in which dispersal and extinction along branches cause range expansions and reductions, respectively. At cladogenetic events, the DEC model allows diversification either within a single ancestral range or within one area of a larger ancestral range or between a single area and the remainder of the ancestral range [116], [117]. The DEC model is similar to the DIVA (*Dispersal-Vicariance*) model [118], [119] and it can be perceived as a parametric, extended version of DIVA [120].

We considered the water isotherm of 25°C and vast intercontinental open ocean regions as frontiers to delimit regions needed for the ancestral area reconstruction analysis. The 12 geographical regions are (Fig. 1B): East Pacific (EP), West Atlantic (WA), East Atlantic (EA), Indo-West Pacific (IWP), Ponto Caspian system (PC), Northwest Atlantic (NA), Northeast Atlantic (NE), North Pacific (NP), south South America (SS), South Africa (SA), South Australia (AU) and New Zealand (NZ). The degree of endemism in each of these 12 regions is elevated with few species or genera distributions overlapping more than one of these regions; except within some junction zones between two regions.

To significantly reduce the computation time in Lagrange, we have pruned several biogeographically non-informative clupeoid taxa from the maximum clade credibility tree (Fig. 3). We therefore kept only one species of the following genera, each endemic to the IWP region: *Tenualosa*, *Clupeichthys*, *Clupeoides*, *Sundasalanx*, *Coilia*, and *Stolephorus*. We also reduced the number of species of the well-defined clade of Central African freshwater tribe Pellonulini to two (*Pellonulla vorax* and *Potamothrissa acutirostris*), and of the two New Zealand endemic *Sprattus* to one (*S. antipodum*). We finally deleted the species *Engraulis encrasicholus* due to its very high genetic proximity to *Engraulis japonicus*. Consequently, the number of terminal twigs is reduced to 54 and most of terminal twigs were assigned to one of the pre-defined 12 regions based on the species distributions established in Whitehead [16] and Whitehead et al. [17]. Multi-region coding was used for few terminal taxa representing species-groups having distributions spanning two or more pre-selected regions: *Alosa alosa* belongs to a clade that includes species from Northeast Atlantic, Northwest Pacific and Ponto-Caspian regions [121], [122] (coding: NA+NE+PC); the genus *Brevoortia* (including *B. tyranus*) is distributed in Northwest, West and South Atlantic regions [123], [124] (coding: WA+NA+SS). *Dorosoma cepedanium* is a member of the subgenus *Dorosoma* that includes both East Pacific and West Atlantic species [16] (coding: EP+WA). *Sardinella maderensis* and *Sardinella albella* belong to the subgenus *Clupeonia* which has a broad tropical distribution, including the Indo-West Pacific region, and the East and West Atlantic regions [16] (coding: IWP+WA+EA). The pristigasterid *Pellona* occurs in the Indo-West Pacific and West Atlantic regions along with *Ilisha* (excluding *Ilisha Africana*); *Ilisha*’s distribution furthermore extends to the East Pacific region (coding: IWP+WA and IWP+WA+EP, respectively). The warm-tropical genus *Etrumeus* is atypical within the Clupeoidei because it includes several allopatric species worldwide distributed [14] (coding: IWP+WA+EP+NP+NA+SA+AU). New World anchovies (*Anchiovella* and *Lycengraulis*) are distributed in West Atlantic and East Pacific regions (coding: WA+EP). Finally, the genus *Sardinops* and the “group 1” of the genus *Engraulis*–as defined by Whitehead et al. [17]–are distributed in no less than five and six temperate regions, respectively (coding: NP+NA+SA+SS+AU and NP+NA+NE+SA+SS+AU, respectively). Because the maximal range size is limited to three units (see below) in our analysis, we randomly chose three regions among all possible regions for *Etrumeus*, *Sardinops* and *Engraulis*.

We set up a restriction on the maximal ancestral range sizes, thus they can’t span more than three possible units because the distributions of most of the species and genera of extant Clupeoidei do not span more than three regions (and often they are restricted to only one region). Default options of other parameters were selected.

## Supporting Information

Figure S1 **Bayesian (50% majority rule consensus) tree of the Clupeoidei from the analysis of the partitioned mitogenomic dataset (using the software MrBayes** [**101]**, [**102**] **).** Branch lengths are proportional to the number of substitutions per nucleotide position (scale bar = 0.05 substitutions). Numbers at nodes are posterior Bayesian probabilities if <1. The tree is rooted with *Coregonus lavaretus* and *Esox lucius*. Abbreviation: *C.*, *Chirocentrus*.
(TIF)

Table S1 **List of the 82 species included in this study.**
(DOCX)
