# Supplementary figures and images for: Mitogenomic Evidence for an Indo-West Pacific Origin of the Clupeoidei (Teleostei: Clupeiformes)
Source: PLoS One. 2013 Feb 19;8(2):e56485. doi: 10.1371/journal.pone.0056485 (PMC3576394; doi:10.1371/journal.pone.0056485)

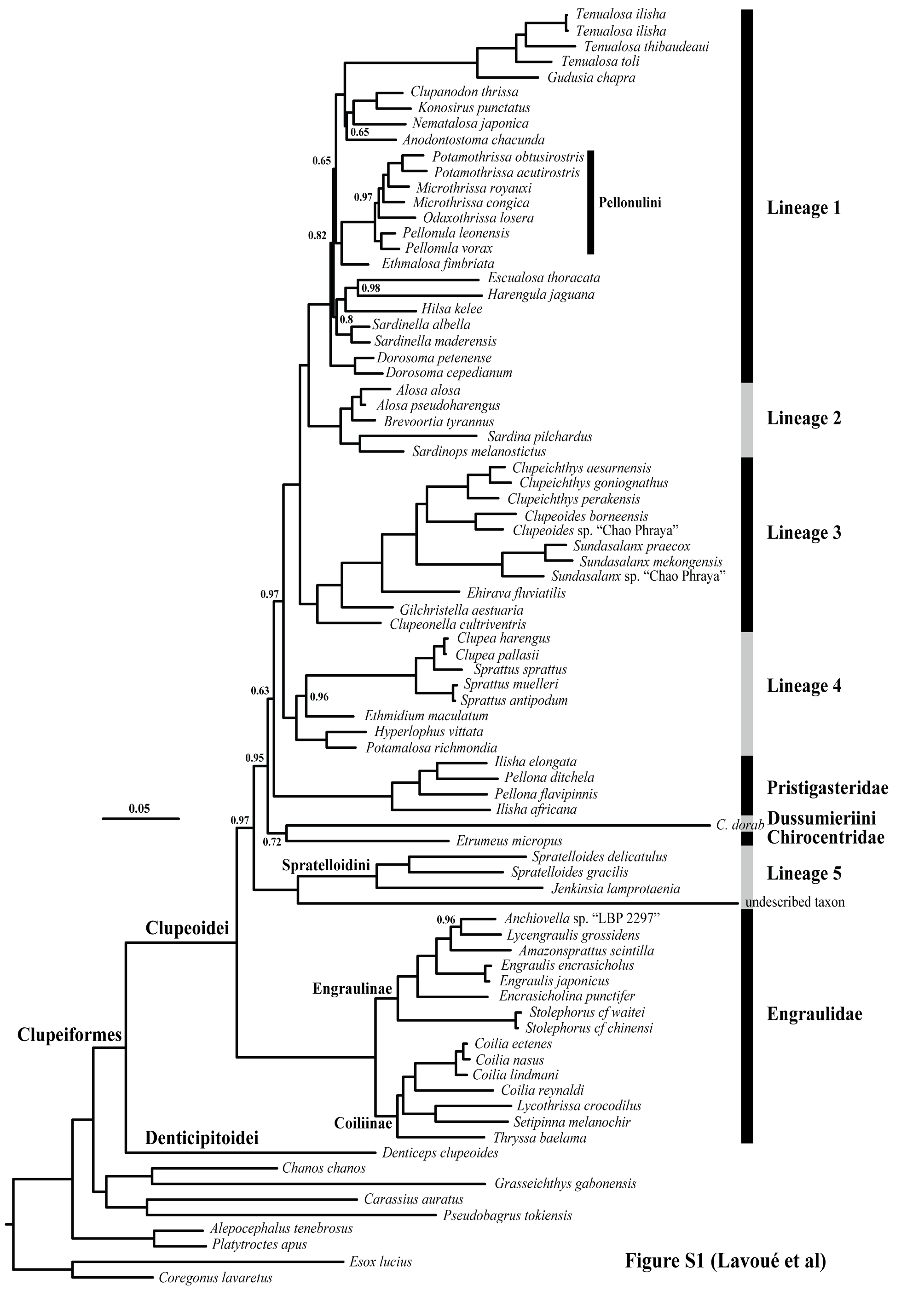

Supplement: Figure S1 — Bayesian (50% majority rule consensus) tree of the Clupeoidei from the analysis of the partitioned mitogenomic dataset (using the software MrBayes [101] , [102] ). Branch lengths are proportional to the number of substitutions per nucleotide position (scale bar = 0.05 substitutions). Numbers at nodes are posterior Bayesian probabilities if <1. The tree is rooted with Coregonus lavaretus and Esox lucius. Abbreviation: C., Chirocentrus. (TIF) [file pone.0056485.s001.tif]
