# Supplementary material for: Mitogenomic Evidence for an Indo-West Pacific Origin of the Clupeoidei (Teleostei: Clupeiformes)
Source: PLoS One. 2013 Feb 19;8(2):e56485. doi: 10.1371/journal.pone.0056485 (PMC3576394; doi:10.1371/journal.pone.0056485)
Supplement: Table S1 — List of the 82 species included in this study. (DOCX) [file pone.0056485.s002.docx]

**Table S1.** List of species examined in this study. Classification follows Nelson [[1](#_ENREF_1)] with the exception of Otocephala, which groups Clupeiformes, Alepocephaliformes, Gonorynchiformes and Otophysi [[2](#_ENREF_2),[3](#_ENREF_3)].

| **Classification** | | **Species** | **Origin** | **Accession Nos.** | **Reference** |
| --- | --- | --- | --- | --- | --- |
| Otocephala | |  |  |  |  |
| Order Clupeiformes | |  |  |  |  |
| Family Denticipitidae | | *Denticeps clupeoides* Clausen | Bénin, West Africa | AP007276 | [[4](#_ENREF_4)] |
| Family Clupeidae | Subfamily: | *Sardinops melanostictus* (Temminck & Schlegel) | Japan, Northwest Pacific | AB032554 | [[5](#_ENREF_5)] |
|  | Clupeinae | *Clupea pallasii* Valenciennes | Japan, Northwest Pacific | AP009134 | [[6](#_ENREF_6)] |
|  |  | *Clupea harengus* Linnaeus | North Atlantic | AP009133 | [[6](#_ENREF_6)] |
|  |  | *Sprattus sprattus* (Linnaeus) | North Atlantic | AP009234 | [[6](#_ENREF_6)] |
|  |  | *Sprattus muelleri* (Klunzinger) | South Island, New Zealand | **AP011607** | **This study** |
|  |  | *Sprattus antipodum* (Hector) | South Island, New Zealand | **AP011608** | **This study** |
|  |  | *Escualosa thoracata* (Valenciennes) | Bangkok, Thailand | **AP011601** | **This study** |
|  |  | *Clupeonella cultriventris* (Nordmann) | Caspian Sea | **AP009615** | **This study** |
|  |  | *Harengula jaguana* Poey | West Atlantic | **AP011592** | **This study** |
|  |  | *Sardinella* *albella* "STIN" (Valenciennes) | Madagascar | **AP011605** | **This study** |
|  |  | *Sardinella maderensis* (Lowe) | Near Dakar, Sénégal | AP009143 | [[6](#_ENREF_6)] |
|  |  | *Sardina pilchardus* (Walbaum) | Europa | AP009233 | [[6](#_ENREF_6)] |
|  | Alosinae | *Ethmalosa fimbriata* (Bowdich) | Near Dakar, Sénégal | AP009138 | [[6](#_ENREF_6)] |
|  |  | *Brevoortia tyrannus* (Latrobe) | North America | AP009618 | [[7](#_ENREF_7)] |
|  |  | *Ethmidium maculatum* (Valenciennes) | East Pacific, South America | **AP011602** | **This study** |
|  |  | *Tenualosa ilisha* "CL10" (Hamilton-Buchanan) | Calcutta, India | **AP011610** | **This study** |
|  |  | *Tenualosa ilisha* "CLT7" (Hamilton-Buchanan) | Bangkok, Thailand, 2008 | **AP011611** | **This study** |
|  |  | *Tenualosa thibaudeaui* "TENUA" (Durand) | Vientian, northern Laos | **AP011604** | **This study** |
|  |  | *Tenualosa toli* "CL03" (Valenciennes) | Calcutta, India | **AP011600** | **This study** |
|  |  | *Gudusia chapra* "CL04" (Hamilton-Buchanan) | Calcutta, India | **AP011603** | **This study** |
|  |  | *Hilsa kelee* "CLT6" (Cuvier) | Bangkok, Thailand, 2008 | **AP011613*** | **This study** |
|  |  | *Alosa alosa* (Linnaeus) | Vilaine River, France | AP009131 | [[6](#_ENREF_6)] |
|  |  | *Alosa pseudoharengus* (Wilson) | North America | AP009132 | [[6](#_ENREF_6)] |
|  | Dussumeriinae | *Spratelloides delicatulus* (Bennett) | Japan | AP009144 | [[6](#_ENREF_6)] |
|  |  | *Spratelloides gracilis* (Temminck & Schlegel) | Japan | AP009145 | [[6](#_ENREF_6)] |
|  |  | *Etrumeus micropus* (Temminck & Schlegel) | Japan | AP009139 | [[6](#_ENREF_6)] |
|  |  | *Jenkinsia lamprotaenia* (Gosse) | West Africa | AP006230 | [[8](#_ENREF_8)] |
|  | Dorosomatinae | *Dorosoma petenense* (Günther) | North America | AP009136 | [[6](#_ENREF_6)] |
|  |  | *Dorosoma cepedanium* (LeSueur) | North America | DQ536426 | [[9](#_ENREF_9)] |
|  |  | *Anodontostoma chacunda* (Hamilton-Buchanan) | Kosamui, Thailand, 2004 | **AP011614*** | **This study** |
|  |  | *Konosirus punctatus* (Temminck & Schlegel) | Tokyo, Japan, 2007 | **AP011612** | **This study** |
|  |  | *Clupanodon thryssa* (Linnaeus) | Northwest Pacific | JX075099 | [15] |
|  |  | *Nematalosa japonica* Regan | Okinawa, Japan, 2004 | AP009142 | [[6](#_ENREF_6)] |
|  | Pellonulinae | *Pellonula leonensis* Boulenger | Ouémé R., Bénin, 2003 | AP009232 | [[6](#_ENREF_6)] |
|  |  | *Pellonula vorax* Regan | Nkomi R., Gabon, 2001 | AP009231 | [[6](#_ENREF_6)] |
|  |  | *Potamothrissa obtusirostris* "CLOD" (Boulenger) | Lower Congo, 2006 | **AP011599** | **This study** |
|  |  | *Potamothrissa acutirostris* "AF3" (Boulenger) | Lower Congo, 2006 | **AP011597** | **This study** |
|  |  | *Odaxothrissa losera* "AF1" Boulenger | Lower Congo, 2006 | **AP011595** | **This study** |
|  |  | *Microthrissa royauxi* "AF2" Boulenger | Lower Congo, 2006 | **AP011596** | **This study** |
|  |  | *Microthrissa congica* "AF4" (Regan, 1917) | Lower Congo, 2006 | **AP011598** | **This study** |
|  |  | *Clupeichthys aesarnensis* "CLAE" Wongratana | Chao Phraya R., Thailand | **AP011584** | **This study** |
|  |  | *Clupeichthys perakensis* "CLPE" (Herre) | Thailand | **AP011585** | **This study** |
|  |  | *Clupeichthys gogniognathus* "CORI" Fowler | Chao Phraya R., Thailand | **AP011589** | **This study** |
|  |  | *Clupeoides borneensis* "CLBO" Bleeker | Chao Phraya R., Thailand | **AP011586** | **This study** |
|  |  | *Clupeoides* sp. "Chao Phraya" (CLF3) | Chao Phraya R., Thailand | **AP011587** | **This study** |
|  |  | *Ehirava fluviatilis* "EHRI" Deraniyagala | India | **AP011588** | **This study** |
|  |  | *Gilchristella aestuarius* "GIAE" (Gilchrist) | Kariega estuary? South Africa [catalog number: SAIAB46983] | **AP011606** | **This study** |
|  |  | *Potamalosa richmondia* (Macleay) | Camden Haven River, Australia [voucher: I.31259-001] | **AP011594** | **This study** |
|  |  | *Hyperlophus vittatus* (Castelnau) | Western Port, Rhyll, Australia [voucher: NMV A 26036-005] | **AP011593** | **This study** |
|  | *Incertae sedis* | New Clupeid (Yoshino et al, in prep) | Philippines | AP009496 | [[10](#_ENREF_10)] |
| Family Engraulidae | | *Engraulis japonicus* Temminck & Schlegel | Japan | AB040676 | [[11](#_ENREF_11)] |
|  | | *Engraulis encrasicolus* (Linnaeus) | Northeast Atlantic | AP009137 | [[6](#_ENREF_6)] |
|  | | *Coilia nasus* Temminck & Schlegel | Japan | AP009135 | [[6](#_ENREF_6)] |
|  | | *Coilia ectenes* Jordan & Seale | China | JX625133 | [16] |
|  | | *Coilia lindmani* Bleeker | Lake Tonle Sap, Cambodia | AP011558 | [[7](#_ENREF_7)] |
|  | | *Coilia reynaldi* "CL15" Valenciennes | Calcutta, India | AP011559 | [[7](#_ENREF_7)] |
|  | | *Lycothrissa crocodilus* (Bleeker) | Lake Tonle Sap, Cambodia | AP011562 | [[7](#_ENREF_7)] |
|  | | *Setipinna melanochir* (Bleeker) | Lake Tonle Sap, Cambodia | AP011565 | [[7](#_ENREF_7)] |
|  | | *Thryssa baelama* (Forsskål) | Indonesia | AP009616 | [[7](#_ENREF_7)] |
|  | | *Stolephorus cf chinensis* "STTh" | Bangkok, Thailand | AP011566 | [[7](#_ENREF_7)] |
|  | | *Stolephorus cf waitei* "CL14" | Calcutta, India | AP011567 | [[7](#_ENREF_7)] |
|  | | *Encrasicholina punctifer* Fowler | Marianne Trench, Pacific | AP011561 | [[7](#_ENREF_7)] |
|  | | *Anchiovella* sp. "LBP 2297" | South America | AP011557 | [[7](#_ENREF_7)] |
|  | | *Lycengraulis grossidens* (Agassiz) | South America | AP011563 | [[7](#_ENREF_7)] |
|  | | *Amazonsprattus scintilla* Roberts | South America | AP009617 | [[7](#_ENREF_7)] |
| Family Chirocentridae | | *Chirocentrus dorab* (Forsskål) |  | AP006229 | [[8](#_ENREF_8)] |
| Family Pristigasteridae | | *Ilisha elongata* (Bennett) | Japan | AP009141 | [[6](#_ENREF_6)] |
|  | | *Ilisha africana* Bloch | East Atlantic | AP009140 | [[6](#_ENREF_6)]) |
|  | | *Pellona flavipinnis* (Valenciennes) | South America | AP009619 | [[7](#_ENREF_7)] |
|  | | *Pellona ditchela* "PRIS" Valenciennes | Bangkok, Thailand, SL | **AP011609** | **This study** |
| Family Sundasalangidae | | *Sundasalanx mekongensis* Britz & Kottelat | Mekong R., Cambodia | AP006232 | [[8](#_ENREF_8)] |
|  | | *Sundasalanx praecox* "SUN2" Roberts | Thailand, | **AP011591** | **This study** |
|  | | *Sundasalanx* sp. "Chao Phraya" (SUN1) | Bangkok, Thailand | **AP011590** | **This study** |
| Order Gonorynchiformes | |  |  |  |  |
| Family Chanidae | | *Chanos chanos* (Forsskål) |  | AB054133 | [[12](#_ENREF_12)] |
| Family Kneriidae | | *Grasseichthys gabonensis* Géry |  | AP007277 | [[4](#_ENREF_4)] |
| Order Cypriniformes | |  |  |  |  |
| Family Cyprinidae | | *Carassius auratus* (Linnaeus) |  | AB006953 | [[13](#_ENREF_13)] |
| Order Siluriformes | |  |  |  |  |
| Family Bagridae | | *Pseudobagrus tokiensis* Döderlein |  | AB054127 | [[12](#_ENREF_12)] |
| Order Alepocephaliformes | |  |  |  |  |
| Family Alepocephalidae | | *Alepocephalus tenebrosus* Gilbert |  | AP004100 | [[2](#_ENREF_2)] |
| Family Platytroctidae | | *Platytroctes apus* Günther |  | AP004107 | [[2](#_ENREF_2)] |
| Euteleostei | |  |  |  |  |
| Protacanthopterygii | |  |  |  |  |
| Family Salmonidae | | *Coregonus lavaretus* (Linnaeus) |  | AB034824 | [[14](#_ENREF_14)] |
| Family Esocidae | | *Esox lucius* Linnaeus |  | AP004103 | [[2](#_ENREF_2)] |

"*", partial mitogenomic sequences

**References cited in table S1**

1. Nelson JS (2006) Fishes of the World. New York: John Wiley and Sons. 601 p.

2. Ishiguro NB, Miya M, Nishida M (2003) Basal euteleostean relationships: a mitogenomic perspective on the phylogenetic reality of the "Protacanthopterygii". Mol Phylogenet Evol 27: 476-488.

3. Lavoué S, Miya M, Poulsen JY, Moller PR, Nishida M (2008) Monophyly, phylogenetic position and inter-familial relationships of the Alepocephaliformes (Teleostei) based on whole mitogenome sequences. Mol Phylogenet Evol 47: 1111-1121.

4. Lavoué S, Miya M, Inoue JG, Saitoh K, Ishiguro N, et al. (2005) Molecular systematics of the gonorynchiform fishes (Teleostei) based on whole mitogenome sequences: Implications for higher-level relationships within the Otocephala. Mol Phylogenet Evol 37: 165-177.

5. Inoue JG, Miya M, Tsukamoto K, Nishida M (2000) Complete mitochondrial DNA sequence of the Japanese sardine *Sardinops melanostictus*. Fish Sci 66: 924-932.

6. Lavoué S, Miya M, Saitoh K, Ishiguro NB, Nishida M (2007) Phylogenetic relationships among anchovies, sardines, herrings and their relatives (Clupeiformes), inferred from whole mitogenome sequences. Mol Phylogenet Evol 43: 1096-1105.

7. Lavoué S, Miya M, Nishida M (2010) Mitochondrial phylogenomics of anchovies (family Engraulidae) and recurrent origins of pronounced miniaturization in the order Clupeiformes. Mol Phylogenet Evol 56: 480-485.

8. Broughton RE, Reneau PC (2006) Spatial covariation of mutation and nonsynonymous substitution rates in vertebrate mitochondrial genomes. Mol Biol Evol 23: 1516-1524.

9. Lavoué S, Miya M, Kawaguchi A, Yoshino T, Nishida M (2008) The phylogenetic position of an undescribed paedomorphic clupeiform taxon: mitogenomic evidence. Ichthyol Res 55: 328-334.

10. Inoue JG, Miya M, Tsukamoto K, Nishida M (2001) Complete mitochondrial DNA sequence of the Japanese anchovy *Engraulis japonicus*. Fish Sci 67: 828-835.

11. Ishiguro NB, Miya M, Inoue JG, Nishida M (2005) *Sundasalanx* (Sundasalangidae) is a progenetic clupeiform, not a closely-related group of salangids (Osmeriformes): mitogenomic evidence. J Fish Biol 67: 561-569.

12. Saitoh K, Miya M, Inoue JG, Ishiguro NB, Nishida M (2003) Mitochondrial genomics of ostariophysan fishes: Perspectives on phylogeny and biogeography. J Mol Evol 56: 464-472.

13. Murakami M, Yamashita Y, Fujitani H (1998) The complete sequence of mitochondrial genome from a gynogenetic triploid "ginbuna" (*Carassius auratus langsdorfi*). Zool Sci 15: 335-337.

14. Miya M, Nishida M (2000) Use of mitogenomic information in teleostean molecular phylogenetics: A tree-based exploration under the maximum-parsimony optimality criterion. Mol Phylogenet Evol 17: 437-455.

15. Li M, Zou K, Chen Z, Chen T (2012) Mitochondrial genome of the Chinese gizzard shad *Clupanodon thrissa* (Clupeiformes: Clupeidae) and related phylogenetic analyses. Mitochondr DNA 23: 438-440.

16. Qiao H, Cheng Q, Chen Y, Chen W, Zhu Y (2012) The complete mitochondrial genome sequence of *Coilia ectenes* (Clupeiformes: Engraulidae). Mitochondr DNA (*Early online*).
